# Pyrosequencing of Bacterial Symbionts within *Axinella corrugata* Sponges: Diversity and Seasonal Variability

**DOI:** 10.1371/journal.pone.0038204

**Published:** 2012-06-12

**Authors:** James R. White, Jignasa Patel, Andrea Ottesen, Gabriela Arce, Patricia Blackwelder, Jose V. Lopez

**Affiliations:** 1 Nova Southeastern University Oceanographic Center, Dania Beach, Florida, United States of America; 2 Food and Drug Administration Office of Regulatory Science, Division of Microbiology, College Park, Maryland, United States of America; 3 University of Miami Center for Advanced Microscopy and Marine Geology and Geophysics, Rosenstiel School of Marine and Atmospheric Science, University of Miami, Miami, Florida, United States of America; Missouri University of Science and Technology, United States of America

## Abstract

**Background:**

Marine sponge species are of significant interest to many scientific fields including marine ecology, conservation biology, genetics, host-microbe symbiosis and pharmacology. One of the most intriguing aspects of the sponge “holobiont” system is the unique physiology, interaction with microbes from the marine environment and the development of a complex commensal microbial community. However, intraspecific variability and temporal stability of sponge-associated bacterial symbionts remain relatively unknown.

**Methodology/Principal Findings:**

We have characterized the bacterial symbiont community biodiversity of seven different individuals of the Caribbean reef sponge *Axinella corrugata*, from two different Florida reef locations during variable seasons using multiplex 454 pyrosequencing of 16 S rRNA amplicons. Over 265,512 high-quality 16 S rRNA sequences were generated and analyzed. Utilizing versatile bioinformatics methods and analytical software such as the QIIME and CloVR packages, we have identified 9,444 distinct bacterial operational taxonomic units (OTUs). Approximately 65,550 rRNA sequences (24%) could not be matched to bacteria at the class level, and may therefore represent novel taxa. Differentially abundant classes between seasonal *Axinella* communities included Gammaproteobacteria, Flavobacteria, Alphaproteobacteria, Cyanobacteria, *Acidobacter* and *Nitrospira.* Comparisons with a proximal outgroup sponge species (*Amphimedon compressa*), and the growing sponge symbiont literature, indicate that this study has identified approximately 330 *A. corrugata-*specific symbiotic OTUs, many of which are related to the sulfur-oxidizing Ectothiorhodospiraceae. This family appeared exclusively within *A. corrugata*, comprising >34.5% of all sequenced amplicons. Other *A. corrugata* symbionts such as Deltaproteobacteria, *Bdellovibrio,* and *Thiocystis* among many others are described.

**Conclusions/Significance:**

Slight shifts in several bacterial taxa were observed between communities sampled during spring and fall seasons. New 16 S rDNA sequences and concomitant identifications greatly expand the microbial community profile for this model reef sponge, and will likely be useful as a baseline for any future comparisons regarding sponge microbial community dynamics.

## Introduction

Recognition that many biological processes often involve multiple organismal partners continues to grow, yet symbiosis research remains a relatively understudied field – compared to cancer biology or genomics. Symbiosis between eukaryotic hosts and microbes can affect whole organismal (“holobiont”) health, encompasses complex microbial community interactions and can lead to construction of large three-dimensional structures such as coral reefs [1], [2], [3].

Sponges live on many types of reefs and represent the oldest metazoan phylum, having existed since the Cambrian period 500 million years ago [4], [5]. With regard to diverse microbial microcosms, marine sponges can be viewed as a microbial niche, incubator and nurturing host par excellence. In some sponge species, microbes may reach over 50% of the total system biomass [6], [7]. Due to its filter-feeding lifestyle, a 1 kg sponge can filter up to 24,000 L of seawater per day, which will include some bacterioplankton [8], [9], [10]. However, recent “next generation” DNA sequencing data indicate that many of these water column-derived bacteria do not colonize very well [11], perhaps due to the pre-adapted symbiont complexes already present in the sponge mesohyl.

Over the past two decades, the sponge research community has identified a large number of the microbial taxa that reside and appear to be symbiotic within this unique marine invertebrate [12], [13], [14]. Since Wilkinson’s pioneering papers on the culture of sponge-associated microbes, numerous studies have emerged, applying modern molecular tools and culture-independent methods based on 16 S rRNA gene sequences to characterize sponge microbial communities [15], [16], [17], [18], [19], [20]. Recent next generation DNA sequencing studies have shown up to 3000 microbial operational taxonomic units (OTUs) across several sponge species, including *Ianthella basta*, *Ircinia ramosa* and *Rhopaloeides odorabile* [11], [21]. Moreover, a sponge-specific bacterial phylum, “Poribacteria”, has been proposed due to its association and presence in several sponge species [22].

In spite of the recent progress, many questions regarding the specific ecological roles and mechanisms of individual microbes or communities within sponge microcosms remain unanswered, partly due to the paucity of robust sponge models. How and why has the sponge-microbial symbiosis system persisted for hundreds of millions of years? How stable are these symbioses even within shorter time frames such as years or decades?

In order to address some of these gaps in knowledge and to expand a model for sponge symbioses, biomedicine and natural products chemistry, the marine sponge *Axinella corrugata* has been chosen for a deep temporal bacterial community profile. *A. corrugata* has wide distribution in the Gulf of Mexico, Florida, and east coast of the United States from Georgia to North Carolina [23], [24], [25] (and see http://porifera.lifedesks.org/pages/1080). This sponge is found in the southern Caribbean along the Venezuelan and Colombian coasts, as well as off Curacao, Dominican Republic, and the Bahamas [23]. *A. corrugata* also produces the secondary metabolite ‘stevensine’, an alkaloid metabolite that has function as a protective measure to deter predatory reef fishes [26], as well as antitumor and weak antimicrobial properties [27], [28]. Previous research has characterized its cell culture, aquaculture, sponge-specific genetic markers, and begun the characterization of *A. corrugata-*specific microbial communities [24], [29], [30], [31]. Although marine microbes do not appear directly responsible for stevensine production [32], the microbial communities within *A. corrugata* likely have important functions for the host.

In this context, the present study encompasses several goals. Firstly we aim to obtain a comprehensive profile of the typical *A. corrugata* bacterial symbiont community by applying high throughput, “next-generation” sequencing methods. Secondly, this effort represents a survey of *in situ* sponge microbial community diversity over time, by testing the null hypothesis that predominant *A. corrugata* microbial symbiont profiles do not change according to seasonal and temperature variations. Third, the data generated from these advanced technologies will be analyzed with state-of-the-art next-generation bioinformatics software and algorithms, including the Quantitative Insights Into Microbial Ecology (QIIME) package and the CloVR virtual machine.

## Results

### Overall Diversity and Taxonomic Composition of Sponge Microbiota

Sequenced 16 S rRNA amplicons were rigorously assessed for quality as well as contaminant and putative chimeras (see *Methods*). Table 1 provides an overview of the results of quality filtering, chimera detection and analysis.

**Table 1:** Overview of the results of quality filtering, chimera detection and analysis for all sponge samples.

Total sponge samples	8
Total raw sequences	300,801
Sequences below length requirement	29,314
Sequences violating homopolymer limit	36
Sequences passing quality filtering	271,451
Putative chimeric reads	5,939
Final high-quality sequence count	265,512
Avg. reads per sample	33,189
Final number of OTUs	9,444
Unique phyla detected	18

**Figure 1:**
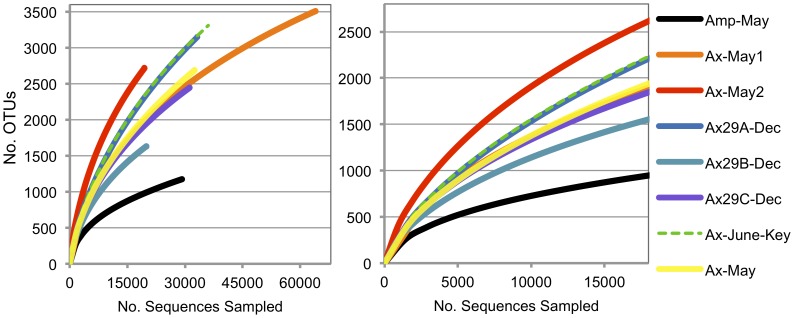
Rarefaction plots of OTU diversity for each sample. The right plot is a subset of the left plot with equal sampling depth across all samples. Significantly fewer OTUs were observed in the *Amphimedon* sample relative to the *Axinella* communities (at equivalent sampling depths, 95% confidence).

**Figure 2:**
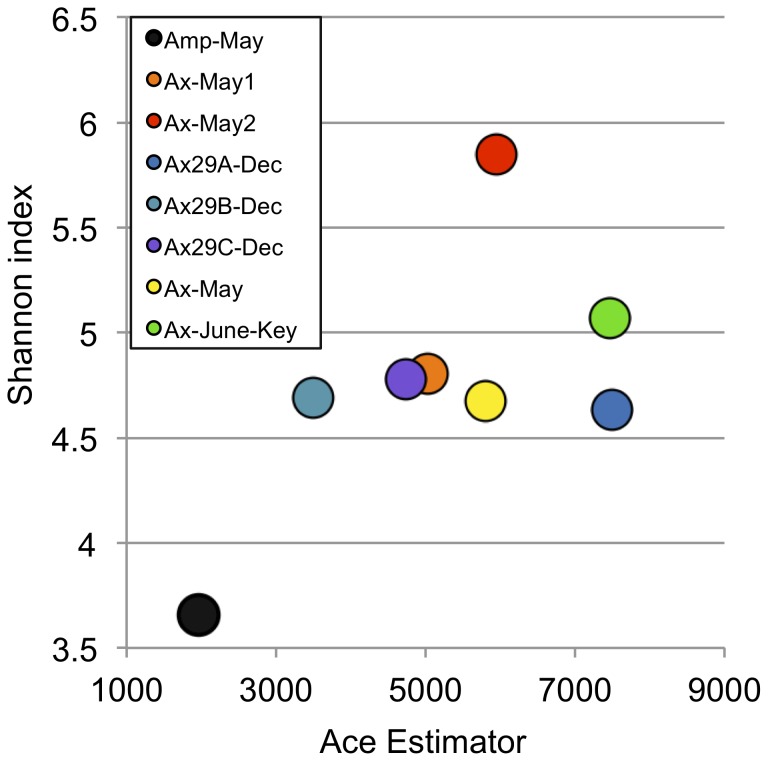
Ace and Shannon diversity measures. To prevent bias due to sampling depth, all samples were first rarefied to 18,000 sequences per sample. The *Amphimedon* community appears less diverse relative to the *Axinella* samples. All *Axinella* samples were significantly more diverse according to Ace and Shannon measures (95% confidence intervals).

To assess the shared diversity among samples, the high-quality chimera-filtered dataset of 265,512 16 S sequence fragments was clustered into species-level OTUs using a 97% pairwise-identity threshold. The average read length across all the sponge samples was 423 bp. A total of 9,444 OTUs were generated, 2,728 and 1,613 of which contained ≥5 and ≥10 sequences, respectively. Figure 1 displays rarefaction plots for each sample. At least 1000 OTUs were observed in each sample, indicating the sponge symbiont community is highly complex. The outgroup *Amphimedon* community (Amp-May) revealed the least overall species-level diversity, significantly less than all other samples (95% confidence). This was supported by Ace and Shannon ecological diversity measures (Fig. 2). Additionally, the Ax-May2 sample resulted in the most diverse OTU structure, significantly more than all other samples (95% confidence). This is interesting given that the associated Ax-May1 sample maintained roughly 30% less OTU diversity (at equivalent sampling depth).

**Figure 3:**
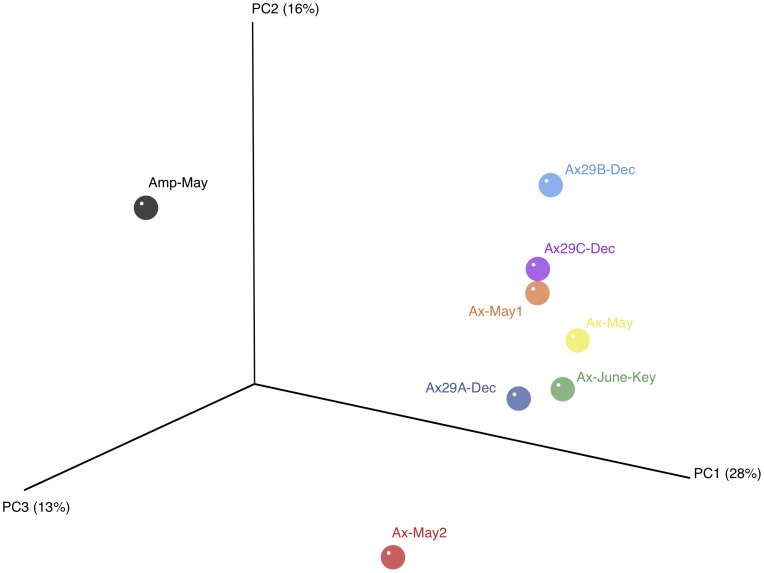
Principal coordinate analysis (PCoA) plot of samples using the unweighted UniFrac distance metric. The variance explained by each principal coordinate axis is shown in parentheses. Datasets were subsampled to equal depth prior to the UniFrac distance computation.

To compare community compositions across samples, we used the unweighted UniFrac metric in QIIME to assess beta-diversity. The principal coordinate analysis plot of the UniFrac distance matrix (Fig. 3) easily distinguishes *Amphimedon* and *Axinella* samples. However the variability among the *A. corrugata* samples is also noteworthy, especially for the highly diverse Ax-May2 sample.

**Figure 4:**
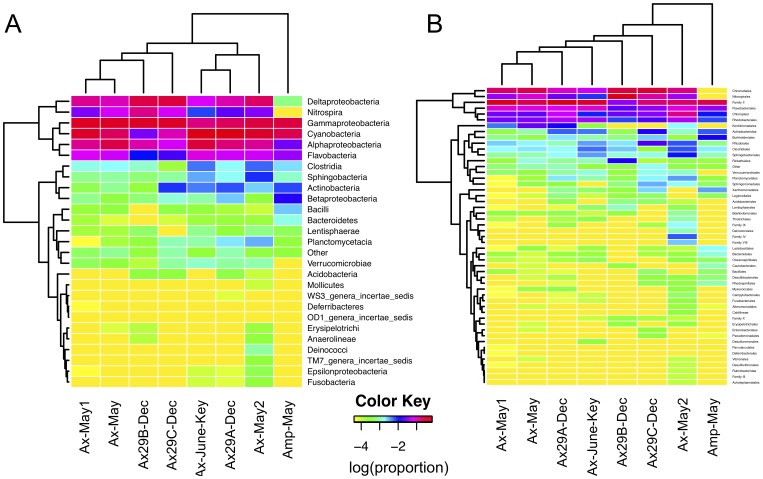
Unsupervised cluster analysis of taxonomic assignments using CloVR. The assignments are either at the class (A) or order (B) levels. Values in the heatmap reflect the log of the relative abundance within each sample (e.g. -1 ∼ 10%, -2 ∼ 1%). We observe that *Amphimedon* is consistently an outlier relative to the other samples, in part due to a lack of Deltaproteobacteria species and *Nitrospira* and a larger abundance of Betaproteobacteria, *Bacilli*, and *Bacteroidetes*.

Representative sequences from each OTU were assigned to a taxonomic lineage using the RDP Bayesian classifier. Examining reads with phylum-level assignments, we observed that Cyanobacteria and Proteobacteria dominated all samples. Among the latter, Gamma- > Delta- > Alphaproteobacteria in overall abundance, while Epsilonproteobacteria were found in trace numbers. Figure 4 displays results of unsupervised clustering of samples based on relative abundances of taxonomic groups at the class and order levels. The distinction between the *Amphimedon* and *Axinella* communities is immediately recognized by the relatively low levels of *Nitrospira*, Deltaproteobacteria and high levels of Betaproteobacteria and *Bacilli* in *Amphimedon*. The single geographical outlier sample from Summerland Key (Ax-June-Key) exhibited microbial profiles that mostly conformed to Broward county *A. corrugata* samples, except for taxa such as a) *Nitrospira* and b) *Clostridia* and *Sphingobacteria* that appear at lower and elevated levels in Summerland Key, respectively (Fig. 4).

Examining which OTUs were shared between communities, we discovered 377 OTUs were present in all seven *A. corrugata* samples, 331 of which were specific to *A. corrugata* (i.e. not observed in the *Amphimedon* sample). These *A. corrugata* specific OTUs spanned multiple phyla including: Proteobacteria (130), *Bacteroidetes* (3), Cyanobacteria (5), and *Nitrospira* (6). *Nitrospira* comprised a ubiquitous and diverse group within *A. corrugata* at around 2% total composition. Interestingly *Nitrospira* sequences did not appear in the single *Amphimedon* sample, as it had in a previous study [33].

One prevalent OTU assigned to Ectothiorhodospiraceae (OTU 118) had at least 850 observations in all *Axinella* samples, but none in *Amphimedon,* which had zero OTUs assigned to Ectothiorhodospiraceae. Phylogenetic analysis of selected bacterial groups such as OTU 118 was performed to determine intra-clade variation. Fifty to sixty random OTU 118 sequences from each of the *A. corrugata* hosts were analyzed with MEGA, resulting in uncorrected and Kimura-2N corrected mean distances that were <1.0%. This finding included sequences from the Florida Keys sample (Ax-June-Key), indicating high sequence conservation within this clade across geographical distances. Neighbor joining and maximum parsimony reconstructions with up to 63 OTU 118 sequences were generally polytomous, as there were only 51 variable sites out of 521, with 13 of these being parsimony informative (Fig S1).

Strikingly, 187 *A. corrugata -*specific OTUs could not be confidently assigned to any bacterial phylum. On average these unassigned OTUs made up over 36% of 16 S fragments from *A. corrugata* samples. The remaining 46 of the 377 OTUs observed in all seven *A. corrugata* samples, were also observed in the *Amphimedon* sample. These OTUs represented on average 13.9% of 16 S fragments, and over 46% of all sequences observed from the *Amphimedon* community alone (see Table 2). Taxonomic assignments of these OTUs included: 21 *Cyanobacteria*, 7 Proteobacteria, and 10 *Bacteroidetes*, as well as 8 OTUs that could not be confidently assigned to a phylum.

**Figure 5:**
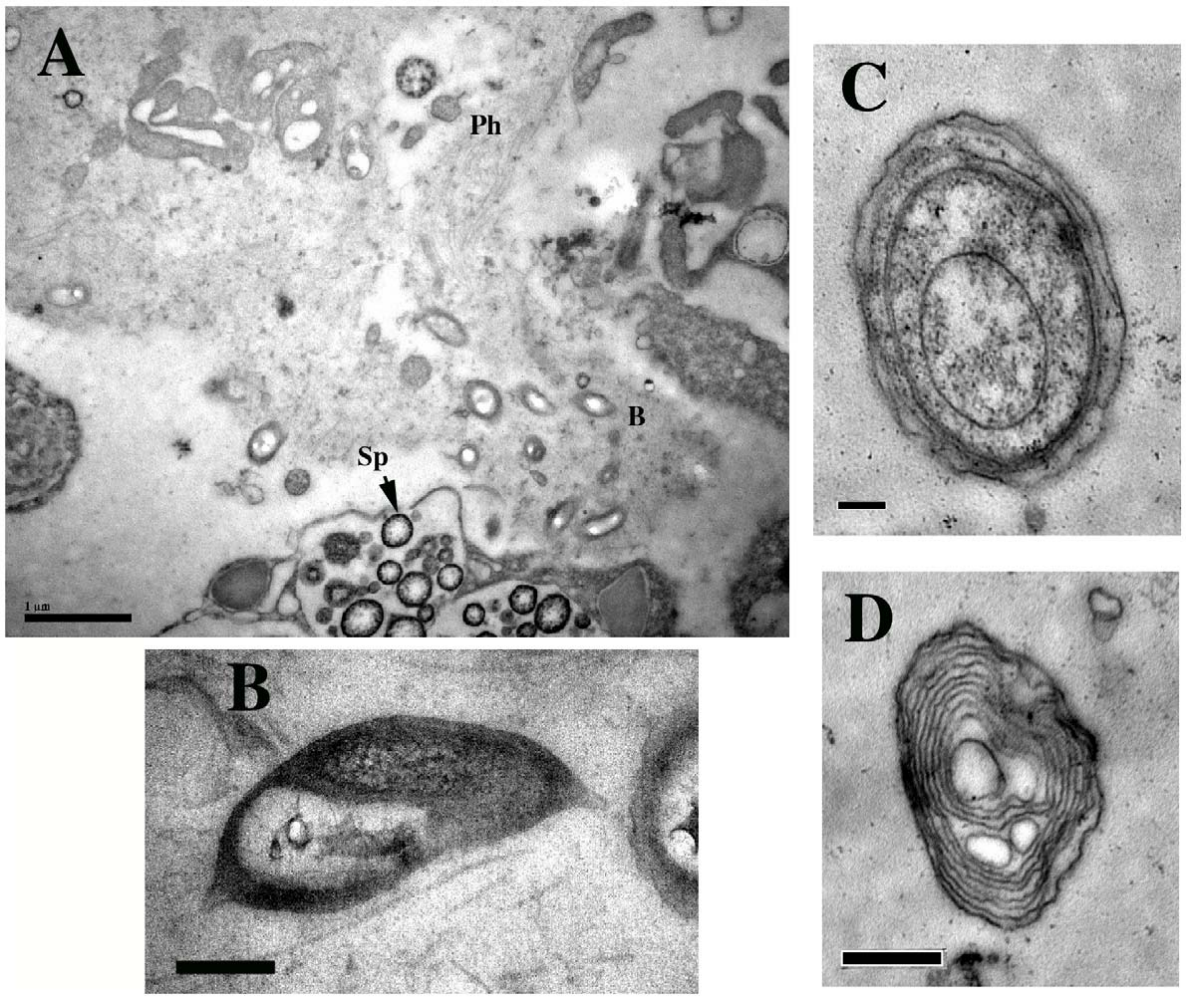
Representative TEM micrographs of *Axinella corrugata* sponge mesohyl. A) Wide angle view showing potentially aggregated bacteria (b), possible phage (Ph) and spicule –forming cells (Sp). Scale bar = 1 µm; B) One of several unidentified pear-shaped bacteria within *Axinella corrugata* sponge mesohyl. Scale bar = 0.2 µm; C) Possible *Cyanobacteria,* Scale bar = 1 µm; D) Possible Ectothiorhodospiraceae microbial symbiont within *Axinella corrugata*. Scale bar = 0.5 µm.

The large number of unidentifiable OTUs led us to create a secondary taxonomic assignment procedure in which we queried all 16 S fragments against the SILVA SSU rRNA database using BLASTN (minimum e-value threshold of 1e^-5^). In the interest of finding the nearest known species for each sequence, we reduced the SILVA database to only references with taxonomic identifications. Using the best-BLAST-hit of each read, we were able to give 99.9% of the sequences a secondary taxonomic assignment at the species level. Table S1 displays the most abundant species assignments in the *A. corrugata* samples. Overall only 18 species were assigned with an average relative abundance greater than 1%, suggesting a substantial number of low frequency members in these communities. We discovered remarkable differences in assignments between the *Amphimedon* and *Axinella* samples, most notably the dominance of purple sulfur bacteria *Ectothiorhodospira sp.* in *A. corrugata* communities, and its virtual nonexistence in *Amphimedon.* Transmission Electron Microscopy (TEM) analyses reveals some cells with multiple lamellar type internal membranes (Fig. 5A and 5D), that appear distinct from possible Cyanobacteria (Fig. 5C). Also notable albeit few in number, ten 16 S rRNA sequences matching to potentially pathogenic *Vibrio* and *Legionella* spp. were detected. Other interesting *A. corrugata*-specific taxa not typically highlighted in previous sponge symbiont surveys include *Parvularcula* sp., *Sedimentiocola-*like endosymbionts of *Ridgeia piscesae.* and iodide-oxidizers, (Table S1). Among the most common Deltaproteobacteria were matches to unidentified clone “Sh765B-TzT-29″.

**Table 2:** Relative abundance of sequences within universally observed OTUs across all sponge samples.

Sample Name	Percentage of sequences in OTUs universally found in all samples
Amp-May	46.25%
Ax-May1	8.04%
Ax-May2	8.65%
Ax-May	7.08%
Ax29A-Dec	14.73%
Ax29B-Dec	0.96%
Ax29C-Dec	3.38%
Ax-June-Key	21.88%

The presence of sulfur-metabolizing bacteria across two time periods alluded to a stable sulfur metabolism, as well as possible alkaline and ultrahaline microhabitats within the *A. corrugata* sponge. A sequence that appeared conspicuous by its low abundance in the current datasets is the sponge-specific taxon Poribacteria [34]. Missing taxa may likely reflect differences in universal rRNA primers applied in specific studies or DNA extraction as well as sponge host (see Discussion).

### Temporal Variability in Community Composition

Although temperatures between May and December timepoints varied by only about 5**°**C in Broward county, other non-temperature related factors could contribute to seasonal differences in S. Florida coastal waters and affect change in community profiles. To compare different taxonomic classes between sample groups, we used the Metastats program at each phylogenetic level (phylum down to OTU assignments). One limitation of the Metastats method is poor estimation of the false discovery rate (FDR) in cases where <100 features are present. As a solution to these cases, we use an earlier approach to estimate the total FDR of a set by Benjamini and Hochberg [35].

We identified 11 differentially abundant class–level groups between spring and fall season *A. corrugata* samples (FDR ∼ 0.1%) (see Table 3). Differentially abundant classes included Gammaproteobacteria, Alphaproteobacteria, Cyanobacteria, *Acidobacter* and *Nitrospira.* Cumulatively, these differentially abundant classes made up over 99% of sequences with taxonomic assignments in both spring and fall *A. corrugata* communities, suggesting potentially high seasonal variability between the dominant bacterial members. Different groups of *Thioalkalivibrio* also seemed to have incongruent patterns between time points. For example *Thioalkalivibrio thiocyanodenitrificans-*like sequences, which made up 1.5% of total observed sequences, appeared more prevalent in December than in May, while *Thioalkalivibrio* sp. K90mix strain had the opposite pattern.

**Table 3:** Differentially abundant taxonomic classes detected between spring and fall *Axinella corrugata* bacterial communities.*

	May		December		
Taxon	mean	std. err.	mean	std. err.	p-value
Alphaproteobacteria	16.97%	0.18%	11.82%	0.17%	7.067E-94
Flavobacteria	6.41%	0.12%	3.37%	0.10%	6.525E-86
Cyanobacteria	26.95%	0.21%	21.06%	0.21%	9.121E-84
Deltaproteobacteria	14.11%	0.16%	19.17%	0.21%	6.908E-83
Gammaproteobacteria	30.38%	0.22%	36.46%	0.25%	2.229E-74
Nitrospira	4.16%	0.09%	6.82%	0.13%	1.784E-62
Actinobacteria	0.11%	0.02%	0.53%	0.04%	7.270E-28
Betaproteobacteria	0.05%	0.01%	0.18%	0.02%	3.085E-07
Clostridia	0.26%	0.02%	0.11%	0.02%	1.323E-06
Sphingobacteria	0.27%	0.02%	0.12%	0.02%	5.520E-06
Acidobacteria	0.00%	0.00%	0.04%	0.01%	2.251E-04

* False discovery rate ∼ 0.01%.

Additionally, in samples from both seasons, a large number of sequences could not be assigned to any phylum (>50%). We examined these unknown groups in more detail by comparing OTU abundances between seasonal samples. Of the 8,000 considered OTUs from *A. corrugata* seasonal samples, 268 were detected as differentially abundant (FDR ∼ 1%); 112 and 156 OTUs were enriched in spring and fall populations, respectively (see Table S2). There were 114 differentially abundant OTUs with no confident assignment to a phylum. Twenty-eight OTUs were assigned to the Cyanobacteria genus GpIIa. Overall these differentially abundant OTUs made up on average 67% and 70% of spring and fall samples, respectively.

### Comparison of Sponge and Environmental Compositions

**Figure 6:**
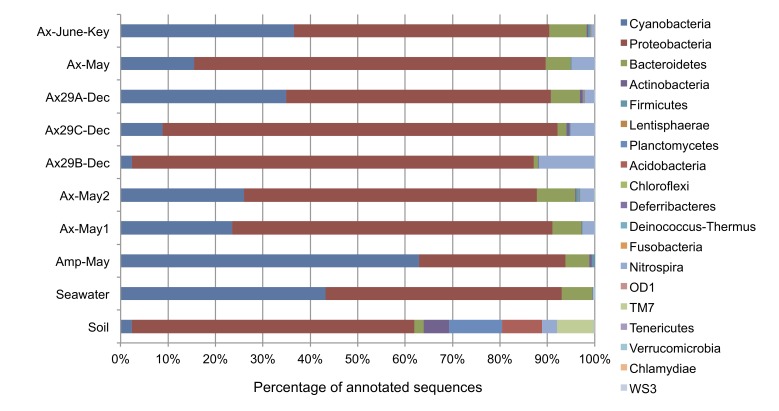
Phylum-level taxonomic assignments of 16 S rRNA sequences for sponge and environmental samples. Overall, phyla such as Proteobacteria, Cyanobacteria, and *Bacteroidetes* tend to dominate both the sponge-specific and water-based microbial communities.

To provide broader comparisons to our dataset, we further sequenced 16 S amplicons from two samples of associated sedimentary and planktonic-based microbial communities, generating a total of 8,905 high-quality pyrosequences. We submitted these sequences to the CloVR-16 S pipeline for taxonomic assignment using the same processing as our original dataset. Figure 6 displays the overall phylum-level distribution of sequence assignments for all samples including the sediment and seawater samples. Across all samples, Proteobacteria was a dominant member, representing 26–48% of sequences from each sample. We observe that the sediment sample has several well-represented phyla that are not abundant in the sponge communities including Planctomycetes (9%), *Acidobacter*ia (7%), and the TM7 candidate division (6%). Phyla present in the sponge communities that were not observed in the seawater or sediment samples included Lentisphaerae and Firmicutes. Given the abundance of *Nitrospira* observations across all *A. corrugata* communities, we expected to recover members of this phylum in the associated environmental samples. Intriguingly, we did discover *Nitrospira* members within the sediment sample (2.5% of sequences), but not a single member in seawater. The lack of *Nitrospira* may be due to its low abundance in the surrounding seawater population (too low given our sequencing depth), but may also suggest an environmental niche shared between the *A. corrugata* microbiome and nearby sediment communities. Class level assignments for the environmental samples appear in Table S4.

Finally, we examined the consistency of the current sponge-associated dataset with that of data from a previously sequenced library of *A. corrugata* microbial symbionts [31]. Querying the current pyrosequenced 16 S rRNA dataset reveal 72,115 sequences that match at least one of the 111 earlier accessions with ≥98% identity along at least 95% of its length. The vast majority (>95%) appear as significant hits to *Axinella*-samples and not *Amphimedon* (see Table S3). Thus, we find stable community membership between earlier *A. corrugata* samples and this study.

## Discussion

The extensive pyrosequencing effort described here reveals that similar microbiomes are harbored within different individual *Axinella corrugata* samples and locales, providing a comprehensive profile of microbial diversity within this unique sponge species. Comparisons between two seasons indicate measureable shifts but an overall stability among most microbial community members. Also, certain class level similarities are seen among the microbial consortia of *A. compressa* and *A. corrugata*, but these different sponge species collected at the same location also have distinct symbiotic communities. This data contributes to the growing database of sponge symbiont biodiversity [12], [14], [17], [21], which in turn provides a baseline parameter for potential future comparisons and measurements of environmental perturbations to sensitive aquatic and marine ecosystems such as coral reefs.

### Sample and Pyrosequencing Platform Considerations

The present study has generated a wide profile of bacterial symbionts of *Axinella corrugata* that spans space and time. Deep DNA sequencing has yielded over 265,000 individual 16 S rRNA reads that surpass previous efforts for a Caribbean sponge by several orders of magnitude. Applying limited Sanger sequencing methods, our previous microbial study of *A. corrugata* compared its microbial profile with a Caribbean coral, *Erythropodium caribaeorum* [31]. In spite of the sampling disparity, consistency can be observed between this earlier study and the present one, such as the prevalence of Gammaproteobacteria. Both present and previous 16S rRNA bacterial surveys highlight the clear taxonomic differences between cultured isolates and culture-independent datasets [18]. Also, sponge microbial community composition patterns follow many previous environmental studies which show predominance of relatively few taxa compared to a majority of low abundance sequences that comprise a “rare biosphere” tail [36]. And although probably present, this study did not attempt to characterize any Archaea.

As part of an initial sequencing strategy with the GS FLX pyrosequencing platform, we opted to sequence different, rather than the same individual sponge through time for the following reasons. First, this approach would maximize the profiling of microbial community diversity and an assessment of intraspecific variation within *A. corrugata*. We realized this would not allow tracking an individual sponge’s change over time, but the approach does shed light on how the species symbiont community may change on average with time. Secondly, the collection method of cutting sponge sections for each sample in our study is invasive and destructive (dissection of a large portion of biomass). Thus taking consecutive samples from the same individual over time may not have resulted in statistical independence. Our primary aim was to characterize the discrete physiological parameter of sponge-symbiont composition–which could be highly correlated to host physiology and homeostasis. However, there could be no certainty as to how the first sampling could affect host health, and thus a second sampling even if months later, compared to a naive, fresh sample of the same species, could possibly be biased. In contrast, when performing longitudinal bacterial survey studies on other organisms (e.g. humans), bacterial samples can be obtained by non-invasive swabbing or sampling fluids and excretions [37].

Although our sampling of non-sponge specimens (seawater and sediments) was partially limited due to costs, preference would have it higher and coincident with sponge sampling. Nonetheless, the growing literature and microbial sequence databases characterize tropical Atlantic coastal and pelagic environmental microbial taxa and can clearly distinguish many from *A. corrugata* microbial communities.” [38].

Furthermore, the present study represents the first phase of a wider, ongoing effort to characterize *in situ* gene expression dynamics within the same samples of *A. corrugata*. For the reasons of tissue destruction and invasiveness mentioned above, different types of bias (disturbance effects) would have to be avoided in mRNA sequence comparisons derived from the same individual sponge.

### Universal rRNA PCR Primers

All culture-independent microbiome studies, including this one, have the goal of capturing the widest microbial diversity characterization possible for each respective target habitat. However, the current pyrosequencing method still depends upon the intended “universality” of the PCR primers used, making the choice of these primers pivotal. (Although the best option for deep sequencing strategies would be complete independence from gene specific primers). We applied two universal eubacterial primers (27F and 533r), previously proven to amplify a wide diversity of the eubacterial spectrum in many past culture-independent studies [39]. These primers span the V2 region which has also been shown to be one of the most phylogenetically informative rRNA regions for eubacteria [40]. In this context, it is curious that this study shows a deficiency of the *Chloroflexi*, since only 4 OTUs were found, and *Chloroflexi* have been shown to be a major taxon across many sponge species [16], [21]. However, previous community profiling with a different universal 16 S rRNA primer pair also did not detect any *Chloroflexi* sequences in multiple *A. corrugata* samples [31], and indicated many *Chloroflexi* sequences occurred more often in deeper rather than shallow water sponges [41]. These results contrast with a recent sponge symbiont pyrosequencing survey by Schmitt *et al,* [21] that applied a modified, slightly more degenerate 533r primer. Their results designate a fairly small number of “core” flora for Phylum Porifera, which included *Chloroflexi* and Proteobacteria, sometimes excluding Poribacteria. Our results re-emphasize the tangible differences between diverse sponge hosts and the additional variables that can affect small subunit rRNA censuses: PCR primer sequences, alternative DNA extraction methods, geographical source or distinct features of host microcosm and identity. We are currently testing alternative 16S rRNA primers that may be established as standard primers for accessing an even wider number of taxa and habitats as part of a burgeoning “earth microbiome” project (http://www.earthmicrobiome.org/) [42], [43].

### The Unique Host Sponge *Axinella corrugata*

*A. corrugata* possesses many interesting traits justifying its elevation as a model sponge. For example, its distribution is fairly widespread across the Western Atlantic, Caribbean and Gulf of Mexico [23], [24]. Secondly, the species has been applied in aquaculture studies [30]. Moreover, marine sponges continue to attract attention due to their production of many chemically diverse marine natural products [44] which still have great potential in pharmacological research [6], [45]. Retention of the ability to produce stevensine in antibiotic-supplemented *A. corrugata* cell culture supported a sponge biosynthetic origin [32]. Stevensine also has weak antimicrobial activity at a concentration of 50 to 200 mg ml^-1^ against certain marine microbial strains, and thus could be a regulator of the microbial community.

As mentioned, there have been long standing efforts to establish permanent *A. corrugata in vitro* cell lines for cell biology research [29], [32]. The characterization of a fairly stable core bacterial community, that includes phototrophic Cyanobacteria, may explain why permanent cell culture of *A. corrugata* remains a goal difficult to attain. It may be likely that the apparently stable and intimate sponge-microbial associations demonstrated in this study, can make permanent axenic growth of pure sponge cell lines difficult to establish *in vitro*.

### *Axinella corrugata* Microbial Symbionts

Although preliminary ultrastructure analysis suggests that *A. corrugata* may be a relatively low microbial abundance (LMA) host, this designation contrasts with high taxonomic diversity observed from the 16 S rRNA data. Unknown taxonomic identities are reflected in the unusual morphologies of several bacteria such as those in Fig. 5B. Its also possible that some specific bacterial species may be relegated to specific locations or structures within the sponge similar to the aggregate formations observed in another local reef sponge, *Agelas tubulata* by FISH [33] but not yet detected by our current TEM surveys.

The finding of a large number of *A. corrugata* symbionts that encompass a wide range of heterotrophs, chemoautolithotrophs. phototrophic *Cyanobacteria* and purple sulfur bacteria adds another interesting dimension to this sponge’s physiology. All characterized *A. corrugata* microbial communities showed no deficiency in *Cyanobacteria* which comprised over 7.2% of all sequences. The transfer of photosynthetic nutrients and byproducts may be vital between the host and these bacteria. *A. corrugata* has been found at depths no lower than 71 m [23], a distribution which still fits well within the photic zone. Curiously, Cyanobacteria seemed to be only sparsely detected in TEM micrographs (Fig. 5A and 5C). One interpretation of this is that the identification of Cyanobacteria from *A. corrugata* samples represented transient cells within the mesohyl or seawater.

Although we do not have definitive evidence for low aeration within the mesohyl, this would be consistent with an anaerobic or microaerophilic environment that favors the sulfur and nitrogen metabolism of many *A. corrugata* microbial community members. Clear presence of multiple sulfur oxidizing and reducing taxa are detected in this dataset: *Desulfovibrio, Thiocystis,* and *Thioalkalivibrio* sp. Extremely haloalkaliphilic, obligate chemolithoautotrophic and sulfur-oxidizing species belonging to genus *Thioalkalivibrio* can efficiently oxidize sulfur in alkaline conditions (∼pH 10).

Among the several Chromotiales taxa found in *A. corrugata,* sequences were found similar to *Halorhodospira halophila (*formerly *Ectothiorhodospira halophila*), which is a motile, alkalophilic, sulfide-oxidizing extreme halophile, whose whole genome has been sequenced (DOE Project ID: 15767). *Halorhodospira halophila* has garnered attention for biotechnological applications due to its production of a) blue sensor Photoactive Yellow Proteins (PYP) and b) hydrogen via photosynthetic pathways [46]. The latter has significance for sustainable energy initiatives [47]. All members of Ectothiorhodospiraceae form and excrete elemental sulfur [48]. *H. halophila* is one of the most halophilic eubacteria of the genus *Halorhodospira* found in hypersaline environments that contain sulfide [49], and similar bacteria are known to oxidize sulfur from natural gas and refineries [50]. An interesting question is how these bacteria survive within sponges growing at normal salinity, as we did not determine whether hypersaline microhabitats exist within the sponge. It is possible that sulfur compounds cycle as potential electron donors which can be used by the various chemotrophic and phototrophic bacteria found in *A. corrugata* communities.

Even if not directly coupled with sulfur, nitrogen metabolism of some *A. corrugata* symbionts, such as *Nitrospira,* probably occurs and is consistent with the evidence of sulfur metabolism. Atmospheric nitrogen fixing symbiotic Cyanobacteria, ammonia-oxidizing Gamma- and Betaproteobacteria and nitrite-oxidizing *Nitrospira* have been previously recovered from sponges and marine habitats [51], [52]. Anaerobic denitrification (reduction of nitrate to nitrogen) is a crucial process in the release of global atmospheric nitrogen which occurs primarily on the seafloor [53] with bacteria as significant contributors. Anaerobic microbial processes such as sulfate reduction have been detected in the sponge, *Geodia barreti* [52]. Genus *Thioalkalivibrio* also includes nitrate-reducers, facultative alkaliphiles and denitrifiers among its nine identified species [54]. *A. corrugata* may be a suitable candidate to study interspecific interactions and rates of complex nitrogen cycling in sponges owing to the presence of *Nitrospira sp., Thioalkalivibrio sp.* and Cyanobacteria.

Another interesting, though rare, taxon found at around 0.06% total abundance in *A. corrugata* communities matches the genus *Rubellimicrobium*, a member of the family Rhodobacteraceae in the *Roseobacter* clade, which is associated with an oil tolerant microbe *Wenxinia marina* [55]. This finding is relevant in the wake of the 2010 BP Deepwater Horizon oil spill and the increased focus on microbial taxa that may be involved in metabolizing polycyclic aromatic hydrocarbons (PAH) and petroleum based hydrocarbons and possible remediation [56], [57].

Many bacterial species are known to give a sponge its color, derived from specific pigments. *A. corrugata*, is a reddish orange hue. Photoacclimation by cyanobacterial symbionts and different phycobiliprotien ratios have also been suspected as a reason for maroon, brown or yellow colors of other sponges. In this context, carotenoid and yellowish pigments are produced by *Parvularcula lutaonensis* and several *Thioalkalivibrio* strains found in *A. corrugata*, respectively [58]. Members of the family Flavobacteriaceae have been found in the current dataset and other pigmented sponges. Many marine taxa belonging to family Flavobacteriaceae are also known to have carotenoid or flexirubin pigments or both which cause a yellow coloration [59].

In the context of the above metabolic considerations, in the future it would be interesting to test hypotheses regarding sponge symbiont interactions and membership in discrete “networks” [60]. Community models can classify organisms according to trophic level or degrees of ecological specialization. Habitat complexity or the apparently high level of metabolic distinctiveness found in some *A. corrugata* symbionts can explain why invasion from transient environmental bacteria may be difficult.

### Physical Habitat of *A. corrugata*

Although a discrete, sponge specific community has been characterized, we cannot discount any transient effects of the local marine habitat. Water quality monitoring is important for ocean and human health, especially in the context of local habitats such as coral reefs and highly populated beach areas [61], [62]. Although we had a statistically low number of environmental samples, many *A. corrugata*-specific taxa did not appear abundant in surrounding seawater and sediments (6,472 and 2,433 16S rRNA sequences, respectively). *Ectothiorhodospiraceae* sequences were not detected in our single seawater sample, but some were observed in Broward county sediment and Key West seawater samples deposited in MG-RAST. Future sponge symbiont profiling may fit into these monitoring schemes, due to possible taxonomic overlap of transient, planktonic microbial taxa.

Understanding the basic functions and activities of a diverse microbial consortium begins with the basic cataloging of relevant taxa. This study now nearly completes this process, and highlights some unique bacterial symbionts which set *A. corrugata* apart from its proximal eukaryotic neighbors. Lastly, the current data establishes a foundation for more in depth functional, metabolic and metagenomic analyses that will further elaborate interactions and networks of a complex microbial microcosm.

## Materials and Methods

### Sponge and Environmental Samples

Sponge samples were collected during the day by SCUBA at the same location off the third reef in the Broward County, Florida shown in Table 4 in December 2009 and May 2010. Three *A. corrugata* samples (designated with “Ax” prefix) each were collected from this site in each season. Additionally, in May 2009, *Amphimedon compressa* (Amp) was collected at the same site and in June 2010, one *Axinella corrugata* (Ax-June-Keys) was collected at Summerland Key, Florida. (Table 4). After collections from the reef, live sponges were placed in a bucket of ambient seawater for a 15 min transport back to the laboratory. At the laboratory,10–20 gm subsamples of each sponge was preserved with each of the following methods: a) placement in RNAlater (Ambion) according to the manufacturer’s directions, b) snap freezing within a plastic storage bag placed in an ethanol-dry ice bath, followed by storage–in a −80°C freezer, and c) placement in a 50 ml conical centrifuge tube with 3 volumes of 75% ethanol, thrice changed at 15, 60 min and 12 hour intervals. The primary goal of these methods was for total RNA (see text), and thus each was more than sufficient for preserving genomic DNA. Environmental samples were obtained at the same Broward reef locations, but at different timepoints than the sponges. Overall, no specific permits were required for the described field studies, since collections did not involve endangered species and did not occur within a designated marine protected area, private reserve or park.

**Table 4:** Collection site and dates for sponge and environmental samples characterized in this study.

Collection Date	Species orsample type	Sample IDnames	Temp (C)	Location	Depth (m)	MG-RAST Nos.
December 2009	*Axinella corrugata* (sponge)	Ax29A-Dec, Ax29B-Dec, Ax29C-Dec	21.1	26 09.104N,80 04.659W	20	4479833.3, 4479834.3, 4479835.3
May 2010	*Axinella corrugata* (sponge)	Ax-May1,Ax-May2,Ax-May	26.6	26 09.104N,80 04.659W	20	4479830.3, 4479831.3, 4479832.3
June 2010	*Axinella corrugata* (sponge)	Ax-June-Key	28.8	24.80N,80.76W	13	4479829.3
May 2009	*Amphimedon compressa* (sponge)	Amp-May	25.0	26 09.104N,80 04.659W	16.7	4479828.3
July 2011	Seawater	BR-4C	29	26 09.618N,80 04.554W	18	4479836.3
December2010	Reef sediment	Sed 2010	21.0	26 09.104N,80 04.659W	9	4479837.3

Sponge taxonomic identifications were confirmed with gene markers [24], morphology and spicule analysis. It should also be reiterated that taxonomic re-designations have synonymized the former species name of *Teichaxinellaxinella morchella* with *Axinella corrugata* [23], [27], [63].

### Genomic DNA Extraction

For total DNA extractions from the sponge, prokaryotic and eukaryotic cells were “squeezed” out of each sponge sample by mincing 3–5 gm sections of tissue in L buffer [10 mM Tris-Cl, pH 8.0; 0.1 M Ethylenediaminetetraacetic acid 487 (EDTA), pH 8.0; 0.5% (w/v) ] [64]. This buffer preserved cell integrity which could be viewed with light microscopy, while DNA degradation was prevented via high EDTA concentrations. When a 2–3 gm cell pellet was obtained, it was processed with a UltraClean® Genomic DNA Isolation Kit (MoBio) according to kit instructions. Purity and concentration of DNA was measured using a NanoDrop 1000 spectrophotometer (Thermo Scientific) and DNA gel electrophoresis. A_260_/A_280_ ratio of approximately 1.8 and clearly visible bands on the gel, confirmed isolation of pure DNA [64]. DNA quality and size of the fragment was visually confirmed using the Agilent 7500 Bioanalyzer DNA chip, which assessed DNA integrity and base-pair length.

### Preparation of DNA for Pyrosequencing

Total sponge genomic DNA was PCR amplified using conserved 16 S SSU rRNA primers that were fused to Roche Fusion primers (Fusion Primer A: 5′CGTA TCGCCTCCCTCGCGCCATCAG3’ and Fusion Primer B: 5′CTATGCGCCTTGCCAG CCCGCTCAG 3′) each having a unique 10 base DNA bar code according to the thermocycling parameters in Rapid Library Preparation Manual. These were attached to universal 16S small subunit (SSU) rRNA primer sequences - Forward primer 27F GTT TGA TCC TGG CTC AG 3' and Reverse primer 533r 5' TTA CCG CGG CTG CTG GCA C 3'. Primers were annealed 55°C for 60 sec, after initial denaturation at 95°C for 300 s, denaturing at 95° for 60s, and extension at 72°C for 60 s (with 29 additional cycles) and final extension at 72° for 300 s. The resulting 16S rRNA fragments spanned about 350–400 bp including hypervariable V1–V3 regions [38]. Quality and quantity of the amplicons was checked using the DNA 7500 Bioanalyzer chip and Fluorescence-based Quantification Assay (Qubit) respectively. DNA sequencing was carried out by using a GS FLX, Roche pyrosequencer (454 Life Sciences, Maryland, USA).

### Bioinformatics

#### Preprocessing

Barcoded multiplex pyrosequences generated using the 454 Titanium platform were initially trimmed for quality using standard the *sff* software tools from Roche/454. Sequences were preliminarily assessed for possible host contaminant from the amplification process by performing a BLASTN [65] search of sequences against the 16 S rRNA homologous gene in *Axinella*; no evidence of contaminant was detected. All sequences in this study have been submitted to MG-RAST, and assigned the following ID numbers: 4479837.3, 4479836.3, 4479835.3, 4479834.3, 4479833.3, 4479832.3, 4479831.3 4479830.3, 4479829.3 and 4479828.3 (Table 4). We have also deposited the raw sequence data in the NCBI Sequence Read Archive under the project accession: SRP010086.

#### Processing

Reads were input to the CloVR-16 S automated pipeline which uses a comprehensive automated protocol for comparative 16 S sequence analysis. Briefly, the CloVR-16 S pipeline employs several popular tools for phylogenetic analysis of 16S rRNA data including: QIIME [66] and Mothur [67] for sequence processing and diversity analysis, the RDP Bayesian classifier [39] for taxonomic assignment, UCHIME (http://www.drive5.com/uchime/) for chimera detection and removal, Metastats [68] for statistical comparisons, and various R scripts for visualization and unsupervised clustering. The CloVR-16 S pipeline initially screens sequences using a minimum length of 100 bp, a maximum homopolymer run requirement of 8 bp, and also removes sequences containing ambiguous base calls. High-quality sequences are subsequently assessed for chimeras using UCHIME with a reference database of 16 S sequences from known species. The resulting chimera-free reads are clustered into OTUs using the UCLUST module from QIIME with a pairwise identity threshold of 97%. OTU representatives are assigned to a phylogenetic lineage using the RDP classifier with a minimum confidence threshold of 80%. Default settings were used in the CloVR analysis. A full description of the CloVR-16 S standard operating protocol is available online at http://clovr.org. [69].

Additional processing of sequence data was performed using the standardized outputs from the CloVR-16 S pipeline. “Rarefied” datasets (with equivalent sampling depths) were generated in QIIME by randomly subsampling 18,000 sequences from each sample in the full dataset. PCoA plots were visualized using the KiNG software package [70]. Unsupervised clustering of taxonomic groups and samples was performed using the Skiff program in CloVR. Within Skiff, relative abundances of taxa within each sample are log-transformed and clustered using a Euclidean distance metric and furthest-neighbor clustering. To detect differentially abundant taxa between May and December sample time points, we used Metastats with default parameters at each phylogenetic level (phylum down to OTU assignments). For comparisons with less than 100 features (e.g. phyla, classes), the false discovery rate was controlled using a method by Benjamini and Hochberg [35].

To assign each 16 S fragment to it’s closest matching known species, BLASTN searches were performed against the SILVA rRNA database (*SSURef_106_tax_silva*) [71] (reduced to reference sequences with full species-level information) with a minimum e-value requirement of 1e-5. Sequences were assigned to the taxonomy of the best BLAST hit under this criterion. Due to their known presence in the sponge holobiont system, uncultured Poribacteria reference sequences were also included in the reduced SILVA database. To assess degrees of relatedness, subsets of the most common bacterial sequences were analyzed phylogenetically using MEGA5 [72].

### Electron Microscopy

Immediately after collection, small sponge sections of 3–6 cm^3^ were fixed in 2% gluteraldehyde sodium caccodylate buffered sea water, posted-fixed in 1% osmium tetroxide, dehydrated in a series of ethanols, and embedded in Spurr™ low viscosity resin. Blocks were then sectioned, stained with lead citrate and uryanl acetate and examined in a JEOL 100X TEM.

## Supporting Information

Figure S1 **Neighbor-joining phylogenetic tree of representative OTU 118 sequences.** The optimal tree with the sum of branch length = 1.95291608 is shown. The percentage of replicate trees in which the associated taxa clustered together in the bootstrap test (500 replicates) are shown next to the branches. The tree is drawn to scale, with branch lengths in the same units as those of the evolutionary distances used to infer the phylogenetic tree. The evolutionary distances were computed using the Maximum Composite Likelihood method and are in the units of the number of base substitutions per site. The rate variation among sites was modeled with a gamma distribution (shape parameter = 1). The analysis involved 34 nucleotide sequences. All positions containing gaps and missing data were eliminated. There were a total of 225 positions in the final dataset. Evolutionary analyses were conducted in MEGA5 [72]. The same topology was observed with maximum parsiomony and minimum evolution reconstructions. Reference sequences for *Thioalkalivibrio* (343202513 -NR_042855.1) and *Methylomicrobium album* (265678936 -NR_029244.1) were included for reference and rooting.
(TIFF)

Table S1 **Representative unique** ***A. corrugata*** **-specific symbionts (as percentages of total).** * - Average occurrence is based only the six Broward county *A. corrugata-* samples
(XLS)

Table S2 **Differentially abundant OTUs identified between May and December** ***A. corrugata*** **samples.** OTUs are ordered by relative abundance of May samples. OTU abundances were input to Metastats using default parameters. A total of 268 were detected as differentially abundant (with a corresponding false discovery rate ∼ 1%). The May samples contained 112 enriched OTUs relative to the December group, while 156 OTUs were relatively enriched in the December population. No confident phylum assignment could be made for 114 of these OTUs using the RDP Bayesian classifier.
(XLS)

Table S3 **Summary of BLAST query matches in current dataset to previously characterized** ***A. corrugata*** **16S rDNA clones.** 72,115 sequences hit at least one previous reference sequence from the set of accessions you wanted with at least 98% identity along at least 95% of their length.
(XLS)

Table S4 **Class level assignments of 16S rRNA sequences.** Assignments were made using the RDP classifier with a minimum confidence threshold of 80%.
(XLS)
