# Supplementary figures and images for: Pyrosequencing of Bacterial Symbionts within Axinella corrugata Sponges: Diversity and Seasonal Variability
Source: PLoS One. 2012 Jun 12;7(6):e38204. doi: 10.1371/journal.pone.0038204 (PMC3373494; doi:10.1371/journal.pone.0038204)

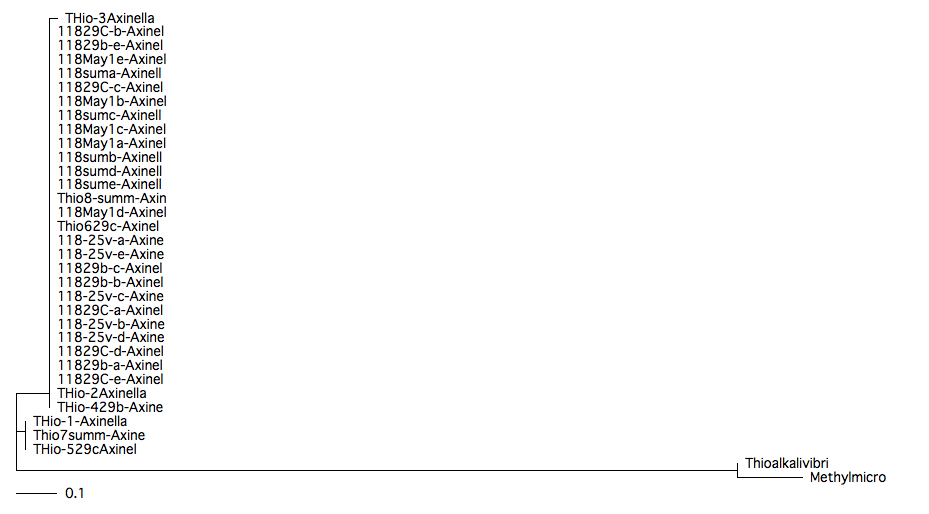

Supplement: Figure S1 — Neighbor-joining phylogenetic tree of representative OTU 118 sequences. The optimal tree with the sum of branch length = 1.95291608 is shown. The percentage of replicate trees in which the associated taxa clustered together in the bootstrap test (500 replicates) are shown next to the branches. The tree is drawn to scale, with branch lengths in the same units as those of the evolutionary distances used to infer the phylogenetic tree. The evolutionary distances were computed using the Maximum Composite Likelihood method and are in the units of the number of base substitutions per site. The rate variation among sites was modeled with a gamma distribution (shape parameter = 1). The analysis involved 34 nucleotide sequences. All positions containing gaps and missing data were eliminated. There were a total of 225 positions in the final dataset. Evolutionary analyses were conducted in MEGA5 [72]. The same topology was observed with maximum parsiomony and minimum evolution reconstructions. Reference sequences for Thioalkalivibrio (343202513 -NR_042855.1) and Methylomicrobium album (265678936 -NR_029244.1) were included for reference and rooting. (TIFF) [file pone.0038204.s001.tif]
